# A randomized controlled trial of a novel mixed monoamine reuptake inhibitor in adults with ADHD

**DOI:** 10.1186/1744-9081-4-24

**Published:** 2008-06-13

**Authors:** Timothy E Wilens, Thorsten Klint, Lenard Adler, Scott West, Keith Wesnes, Ole Graff, Birgit Mikkelsen

**Affiliations:** 1 Clinical Research Program in Pediatric Psychopharmacology, Massachusetts General Hospital & Harvard Medical School, Boston, MA, USA; 2 NeuroSearch A/S, Department of Clinical Development, Denmark; 3 Faculty Practice Offices NYU Medical Center, New York, NY, USA; 4 CNS Healthcare, MD, Orlando, FL, USA; 5 Cognitive Drug Research Ltd, CDR House, Gatehampton Road, Goring-on-Thames, UK

## Abstract

**Background:**

NS2359 is a potent reuptake blocker of noradrenalin, dopamine, and serotonin. The aim of the study was to investigate the efficacy, safety and cognitive function of NS2359 in adults with a DSM IV diagnosis of ADHD.

**Methods:**

The study was a multi-centre, double-blind, randomized placebo-controlled, parallel group design in outpatient adults (18–55 years) testing 0.5 mg NS2359 vs. placebo for 8 weeks. Multiple assessments including computerized neuropsychological evaluation were performed.

**Results:**

There was no significant difference between NS2359 (n = 63) versus placebo (n = 63) on the primary outcome measure reduction in investigator rated ADHD-RS total score (7.8 versus 6.4; p < 0.45). However, in subjects with the inattentive subtype, there were significantly more responders in the NS2359 group compared to placebo (41% versus 7%; p < 0.01). For all secondary variables (ADHD-RS patient rated; The Conners Adult ADHD Scale; The Brown Adult Scale, and CGI-improvement scale) there were no significant differences between the two groups; however, in the inattentive subgroup, the response to treatment was significantly larger than to placebo. NS2359 improved composite factor scores of attention, episodic- and working memory. No serious adverse events were reported with insomnia, headaches and loss of appetite most commonly reported as side effects.

**Conclusion:**

No overall effect of NS2359 was found on overall symptoms of ADHD. There was also a modest signal of improvement in the inattentive adults with ADHD and cognition warranting further exploration using differing doses.

## Background

Attention deficit-hyperactivity disorder (ADHD) is an increasingly recognized and heterogeneous disorder of unclear etiology [1] characterized by core symptoms of hyperactivity, inattentiveness, and impulsivity. The prevalence of ADHD in school-age children is estimated at 6–8% worldwide [2], with symptoms persisting into adulthood in approximately 50% of individuals with childhood onset ADHD [3]. Recent epidemiological data suggest that ADHD occurs in 4.4% of adults in the US [4]. ADHD in adults is associated with academic, employment, and marital difficulties, as well as comorbid psychiatric disorders such as substance abuse, depression, anxiety, and personality disorders [5-7]. Moreover, cost-of-illness data in untreated adults without ADHD show significantly higher cost than in matched adults with ADHD both for medical and societal expenses [8].

The aggregate data also support the concept that ADHD in adults shares many phenotypic and genotypic similarities with the childhood form of the disorder [9]. Due to the phenotypic, genotypic, and pharmacological response similarities between adults and children with ADHD [9] the ethical considerations of exposing children to novel compounds with potential adverse events have led to adults with ADHD being used increasingly for early phase II trials.

Currently, the treatment of ADHD in adults is largely predicated upon use of both stimulant and non-stimulant medications, as well as adjunctive structured psychotherapies [10]. Despite the availability of both FDA-approved and other agents for ADHD, a number of individuals either cannot tolerate, or do not respond to existing compounds necessitating the development of alternative agents.

While the precise etiology of ADHD is unknown continued interest has been focused on the role of the catecholaminergic and nicotinic/cholinergic systems in ADHD. Evidence that dopamine dysfunction plays a role in ADHD comes from findings from studies describing excessive motor activity and cognitive dysfunction and neuroanatomical studies. For instance, compared to controls, differences have been identified in the binding potential of the dopamine transporter protein in adults with ADHD [11-13]. Similarly, candidate genes and functional imaging abnormalities in ADHD point to alterations in areas of the brain rich in dopaminergic/noradrenergic innervations. Along with longstanding associations of cigarette smoking in ADHD [14,15], recent work has shown the efficacy of nicotine and nicotinic analogs in the treatment of ADHD [16,17]. Along those lines, predominately dopaminergic, noradrenergic, and mixed dopaminergic/noradrenergic agents have been shown to have efficacy in treating ADHD [18,19].

One such mixed monoamine reuptake blocker is the novel agent NS2359, which acts by equipotent reuptake blockade across the noradrenalin, dopamine and serotonin transporters. NS2359 has also been shown to enhance the release of acetylcholine in the frontal cortex and hippocampus an effect considered relevant for attentional performance [20,21]. In a previous study NS2359 was found to have positive effects on attention and memory in healthy volunteers [22] and given the high degrees of cognitive dysfunction in ADHD [23], these effects taken together with the mode of action of NS2359 makes it a suitable candidate for treating ADHD. Moreover, the slow entrance of NS2359 into the human brain makes it a candidate for the treatment of ADHD with minimal potential for abuse.

In this paper we report the results of a randomized, double blind, placebo-controlled study of NS2359 in the treatment of adults with ADHD. The primary aim of the study was to investigate the efficacy and safety of NS2359 in DSM IV diagnosed adults with ADHD. The secondary aim was to evaluate the effects of NS2359 on cognitive function, particularly attention, working memory and episodic memory. This study was designed as an exploratory, signal-detection, Phase IIa study to provide proof-of-concept for this novel compound prior to embarking on a larger scale Phase IIb program.

## Methods

### 2.1 Subjects

Patients were recruited by advertisements in local media. Three sites in the US participated in the trial. The study was conducted according to the Declaration of Helsinki and was approved by independent ethics committee or institutional review board at each site. All subjects presented written informed consent. Adult outpatients of either sex (aged 18–55 inclusive) who met DSM-IV criteria for Attention Deficit Hyperactivity Disorder [24] were entered into the study. ADHD needed to be manifested in clinical evaluation and confirmed by structured interview using the Kiddie-Schedule for Affective Disorders (K-SADS-E) adult ADHD module. All subtypes of ADHD were allowed to enroll in the study. Patients were also required to have a CGI Global Severity (GS) score ≥ 4 indicative of moderate impairment. Patients were excluded if they had any clinically unstable medical condition, clinically significant abnormal baseline laboratory values, mental retardation, psychotic disorder, bipolar disorder, current depression (HAM-D > 15), eating disorder, or organic brain disorders including non-febrile seizure disorder. Patients currently (within the past 6 months) known to abuse or to be dependent on any drug, including alcohol or a positive urine drug screen for cocaine, heroin, or marijuana were excluded. Patients were not allowed to use any concurrent medication for the treatment of ADHD. Prohibited treatments were stimulants within 1 week prior to randomization, benzodiazepines, anticonvulsants and lithium for 2 weeks, tricyclic, atypical, and selective serotonin reuptake inhibitor antidepressants within 4 weeks and antipsychotics and monoamine oxidase inhibitors for 8 weeks. Qualified were randomized to receive 0.5 mg NS2359 or placebo orally in the morning for eight weeks without dietary restrictions, and then followed off drug for an additional 4 weeks. The drug was provided as tablets of 0.25 mg or corresponding placebo tablets.

### 2.2 Assessments

The primary efficacy endpoint was the ADHD-rating scale (investigator-rated) which was administered at all study visits. Additional symptom rating scales were used to assess efficacy of secondary endpoints: the ADHD-RS (self-rating scale), the Clinical Global Impression (CGI), the Conners (CAARS) Adult ADHD Scale (self-rating scale) and the Brown Adult Scale (self-rating scale), the latter addressing issues of time management, organization, hierarchical thinking etc. in multiple clinical domains [25].

Additional rating scales were used to evaluate co-occurring mood and anxiety. The Hamilton Depression Scale (HAM-D) was completed by the physician at screening (patients being excluded if the score exceeded 15), baseline (week 0) and at the end of treatment (week 8). The Hamilton Anxiety Scale was also completed by the physician at baseline (week 0) and at the end of treatment (week 8).

The Cognitive Drug Research (CDR) computerized assessment system was used to assess cognitive function in this study. The system has been widely used in clinical research [26,27] and was used previously to identify the positive effects of with NS 2359 on attention and memory in healthy volunteers [22]. The system is a notebook PC based set of tests of attention, working memory and episodic memory. The information is presented on the computer screen and the patient responds on all tasks using a response module containing two buttons, one marked 'NO' and the other 'YES'. The System has numerous parallel forms to allow for repeated testing over hours, weeks or months. The instructions are read to the patient for each task, and the test administrator then initiates each task and monitors the performance of the patient to ensure compliance with the instructions. The data are captured automatically by the computer. The CDR tests selected for this study comprised three attention tests: simple reaction time, digit vigilance and choice reaction time; two working memory tasks: Spatial Working Memory and Numeric Working Memory; and three episodic memory tasks: immediate and delayed word recognition and picture recognition. Each of the tasks is brief, lasting 1–2 minutes, and each test session took about 18 minutes for the patient to complete. The tests have been described in detail previously [26,27]. To familiarize the patients with the tests and overcome training effects, each patient performed the entire battery on two occasions during the screening period. It was then re-administered at baseline (week 0), week 4 and at week 8, and again at the week 12 follow-up, 4 weeks after end of treatment.

Adverse Events (AEs) were by spontaneous report. Standard safety parameters including clinical laboratory tests of blood and urine as well as standard safety parameters (vital signs at each visit, ECGs, physical examination) were completed at various time points throughout the study. Urine was also screened at baseline, week 2, 4 and 6 for qualitative evidence of drugs of abuse.

### 2.3 Statistical Analysis

#### 2.3.1 Sample Size Calculation

The sample size calculation was based on the assumption of a 55% probability for a responder effect (at least 30% improvement/reduction in score for primary endpoint from baseline to week 8) for patients taking active drug, and a 25% probability for effect in placebo, under a 1% significance level and 80% power. The use of a 30% reduction in ADHD symptoms is more conservative than recent trials (25% reduction; [26]) and commensurate with previous work [17,28,29]. This led to a total of 100 patients (50 in each group), but a total of 126 subjects were randomised to allow for an estimated dropout rate of 20%.

The placebo estimate for responding in the ITT population was estimated to be 26.7%, so the placebo assumption of 25%, and hence the above sample size calculation, was justifiable.

#### 2.3.2 Analysis of Clinical Scales

The analysis was based on a repeated measures ANOVA model taking account intrasubject correlation of scores over the 6 study visits. The best choice of model was a linear spatial correlation assumption.

For the systematic variation, the covariates Visit, Treatment and investigator Site, were included as categorical variables with the possibility of mutual second order interaction. The baseline variables Sex (F, M), history of Alcohol (Y, N), Smoker (Y, N, former), Age and Weight were included. Any interactions with these baseline variables were not considered.

In addition, patients were grouped as Inattentive or Combined according to DSM-IV [26] and it was investigated whether treatment interacted with these categories. An extra covariate was included in the ANOVA model to account for an eventual third order interaction between treatment, inattentive/combined and visit. No other changes to the ANOVA models were made. A responder analysis was also conducted for the investigator rated ADHD-RS (response was defined as ≥ 30% improvement. The level of significance was set to 0.05, and all tests were carried out as 2-sided.

#### 2.3.3 Analysis of CDR data

The factor structure of the full CDR task battery has been investigated using Principal Components Analysis [30]. This analysis confirmed the construct validity of the battery by demonstrating that the task variables within theorized cognitive domains of attention, working memory and episodic secondary memory, loaded together on five common factors. Based on this analysis of the individual variables, five combined scores have been derived utilizing all of the individual scores, which loaded on each factor. These combined scores have been used as outcome measures in a variety of studies [27,30].

There are two scores for attention, Power of Attention is the summation of the speed scores from the three attention tests (Simple and choice reaction time, digit vigilance), and reflects the ability to focus attention. Continuity of Attention combines the accuracy scores from choice reaction time and digit vigilance, and reflects the ability to sustain attention, Quality of working memory is a combination of the accuracy scores from the numeric and spatial working memory tasks and reflects the ability to hold information temporarily in memory. Quality of episodic memory combines the accuracy scores from the four episodic memory tasks (immediate and delayed word recall, word and picture recognition) and reflects the ability to store and retrieve episodic information. Speed of Memory is the summation of the speed scores from the two working memory tasks and the two recognition tasks, and reflects the time taken to retrieve information from memory.

A repeated measure ANOVA was conducted on the difference from baseline data using SAS^® ^PROC MIXED. Fixed terms were fitted to the model for treatment, time and treatment*time interaction. A random effect of subjects-within-treatment was fitted to the model. Significance of the treatment*time interaction was tested at the 0.05 level and all testing was two-tailed. Planned comparisons were conducted between the two treatments at each post-dosing time.

The analyses described above have been performed using the SAS system for Windows software version 8.2 or later.

## Results

### 3.1 Subjects

A total of 180 subjects were screened at the 3 study sites. Of the 54 patients not randomized 32 did not meet the study inclusion/exclusion criteria, 14 withdrew their consent to participate in the study and 8 were excluded for other reasons. The 126 subjects included in the study were randomly allocated to treatment groups such that 63 patients received NS2359 and 63 patients received the placebo treatment. All randomized subjects began the treatment protocol.

**Figure 1:**
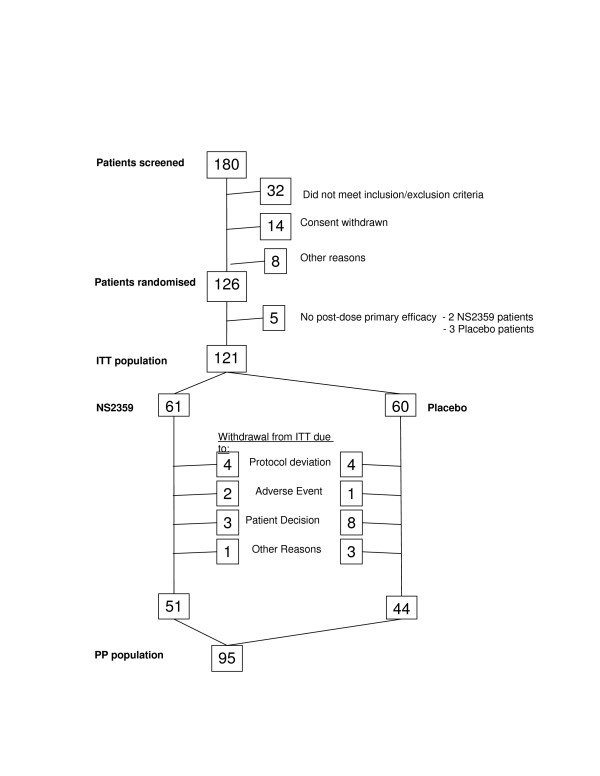
Disposition of Subjects.

A total of 31 subjects either did not complete the 8-week treatment period or had a missing primary endpoint at week 0 and/or week 8. Of these subjects 9 were excluded for a protocol deviation, 3 withdrew due to an adverse event (increased blood pressure (NS2359 treatment); dizziness, irritability and palpitations (NS2359 treatment); dizziness and disorientation (placebo)), 13 decided not to continue and 6 had other reasons for discontinuing the study (4 lost to follow up; 2 in placebo group reported lack of efficacy). A total of 51 and 44 subjects receiving NS2359 and placebo, respectively, were defined as completing the study (Figure 1).

### 3.2 Demographics

Demographic data and baseline values were comparable (not statistically different) between the NS2359 and placebo groups. Data are summarized in Table 1.

**Table 1:** Demographic s and clinical characteristics of Sample

		NS2359	Placebo
Subjects Randomised		63	63
Gender	Male	47 (74.6%)	42 (66.7%)
	Female	16 (25.4%)	21 (33.3%)
Age	18–25	13 (20.6%)	13 (20.6%)
	Median (Range)	35.0 (18.8–54.1)	35.2 (19.0–51.1)
Origin	Caucasian	51 (81.0%)	54 (85.7%)
	African American	4 (6.3%)	3 (4.8%)
	Asian	3 (4.8%)	0 (0.0%)
	Other	5 (7.9%)	6 (9.5%)
Previous Treatment for ADHD	Yes	17 (27.0%)	18 (28.6%)
	No	46 (73.0%)	45 (71.4%)
ADHD Subgroup	Inattentive	17 (27.0%)	29 (46.0%)
	Hyperactive/Impulsive	0 (0.0%)	1 (1.6%)
	Combined	38 (60.3%)	32 (50.8%)
	Not Classified	8 (12.7%)	1 (1.6%)
Weight (kg)	Median (range)	80.3 (54.9–142.9)	78.9 (48.5–133.4)
Height (cm)	Median (range)	173 (152–197)	175 (151–188)
Smoker	Yes	16 (25.4%)	15 (23.8%)
	Former	9 (14.3%)	17 (27.0%)
	No	38 (60.3%)	31 (49.2%)
Alcohol	Yes, Average	53 (84.1%)	58 (92.1%)
Consumption	Yes, Excessive	0 (0.0%)	0 (0.0%)
	No	10 (15.9%)	5 (7.9%)
Hamilton Depression score	Median (Range)	3.0 (0.0–15.0)	4.0 (0.0–12.0)
CGI Severity of Illness	4 – Moderately	30 (47.6%)	29 (46.0%)
	5 – Markedly	28 (44.4%)	31 (49.2%)
	6 – Severely	4 (6.3%)	3 (4.8%)
	7 – Extremely	1 (1.6%)	0 (0.0%)

### 3.3 Efficacy

#### Investigator-rated ADHD-RS

**Figure 2:**
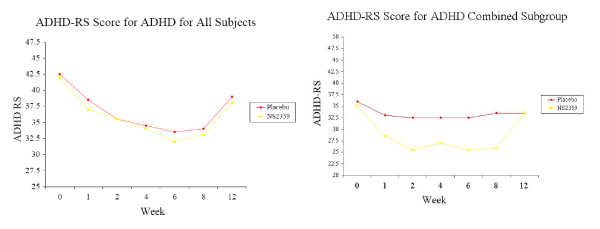
**Mean Investigator rated ADHD-RS Score (ITT Population)** for (a) all Subjects, (b) the ADHD Inattentive Subgroup and (c) the ADHD Combined Subgroup. Active treatment is given week 0 to 8, where week twelve is a follow-up visit.

Overall, there was no significant difference between NS2359 and placebo in the reduction of the primary efficacy variable, the investigator rated ADHD-RS total sum of scores (7.8 (SE 1.3) and 6.4 (SE 1.3), respectively; p < 0.45). Overall, there was no significant difference in the proportion of patients that reduced their ADHD RS score by 30% or more between the NS2359 (33%) and placebo (27%) groups (p = 0.55). In the inattentive subgroup, there was a significantly larger proportion of responders in the NS2359 treatment compared to the placebo treatment (41% and 7%; p < 0.01), although there was no differences in the combined only subgroup (30% and 42%; p = 0.23) Figure 2.

#### Patient self-report ADHD-RS

In the patient-rated ADHD-RS a trend to significance was found between NS2359 and placebo groups (7.2 (SE1.6) and 3.2 (SE 1.2); p = 0.052). While in the inattentive subgroup a significantly greater improvement in ADHD score compared to those on the placebo treatment was observed (8.1 (SE 3.1) and 0.3 (SE1.7); p < 0.05), no significant difference between the 2 groups were observed in the combined group (6.9 (SE 1.8) and 6.3 (SE 1.7); p = 0.82).

#### The Conners (CAARS)

There was no significant difference on The Conners (CAARS) Adult ADHD Scale between medication and placebo groups in the total sum of scores (6.4 (SE 1.5) and 4.7 (SE 1.5); p = 0.42) For the inattentive subgroup there was a significant reduction in score for the NS2359 treatment compared to placebo (7.0 (SE 1.9) and 2.0 (SE 1.5); p < 0.05) that was not observed for the combined subgroup (6.3 (SE 1.9) and 6.9 (SE 2.2); p = 0.86).

#### The Brown Adult Scale (BROWN-AS)

There was no significant difference in The Brown Adult Scale (10.6 (SE 2.8) and 10.8 (SE 2.8); p = 0.97) between the two groups. For the inattentive subgroup there was a significant reduction in score for the NS2359 treatment compared to placebo (15.8 (SE 4.1) and 2.5 (SE 3.1); p < 0.01) that was not observed for the combined subgroup (10.8 (SE 3.6) and 19.8 (SE 4.3); P = 0.11).

#### The Clinical Global Impression (CGI)

There were no significant differences in the CGI-Severity between the NS2359 and placebo groups at any time point (week 8, 3.9 (SD 1.1) and 4.0 (SD 1.1); p = 0.94). However, for the inattentive subgroup treatment with NS2359 gave a significant reduction in the mean severity of illness compared to the placebo treatment at the end of treatment (week 8, 3.3 (SD 0.9) and 4.2 (SD 0.8); p < 0.01) that was not found in the combined subgroup (week 8, 4.0 (SD 1.1) and 3.8 (SD 1.3); p = 0.63).

For the CGI-Improvement assessments there were no significant differences between the NS2359 and placebo groups at any time point (week 8, 3.2 (SD 1.0) and 3.4 (SD 1.1); p = 0.10). However, for the inattentive subgroup significantly greater response was reported on NS2359 compared to the placebo treatment at end point (week 8; 3.0 (SD 1.1) and 3.8 (SD 0.9); p < 0.05) that was again not seen for the combined subgroup at any time point (week 8, 3.2 (SD1.0) and 3.0 (SD 1.2); p = 0.27).

#### The Hamilton Depression and Anxiety Scale

There were no significant changes in depression score in either group over the 8-week study period, although the difference in mean change score was significantly smaller in the placebo group compared with the NS2359 group (Mean HAM-D 3.7(SE 0.4) versus 4.6 (SE 0.4); p = 0.04). Similarly, there were no significant differences in anxiety score between groups over the 8-week study period (Mean-A. 5.7 (SE 0.5) versus 6.3 (SE 0.5); p = 0.12).

#### Computerized Cognitive Assessments

**Figure 3:**
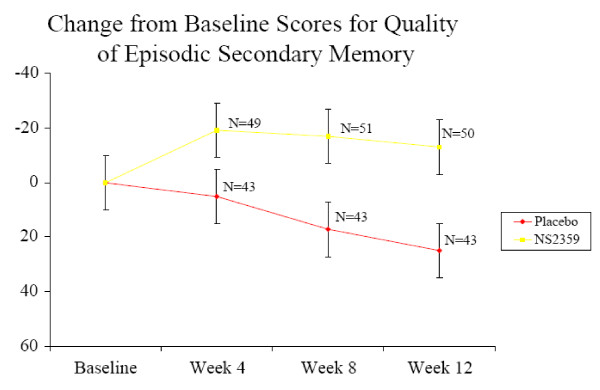
**Change from baseline scores for Quality of Episodic Secondary Memory over the study period (Mean +/- SEM)**. Improvements from baseline are plotted to ascend.

**Figure 4:**
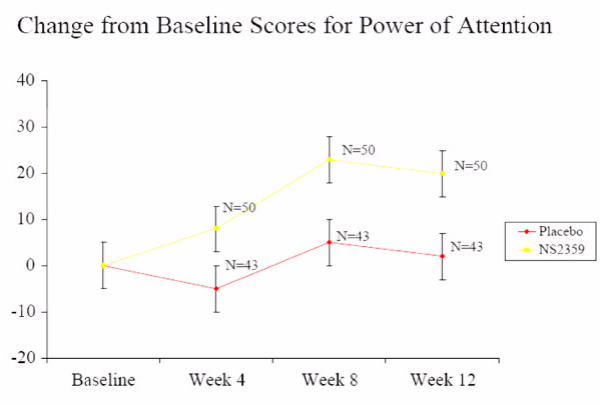
**Change from baseline scores for Power of Attention over the study period (Mean +/- SEM)**. Improvements from baseline are plotted to ascend.

Significant main effects of treatment were found for Power of Attention (p < 0.015) and Quality of Episodic Secondary Memory (p < 0.01). Figures 3 and 4 illustrate that NS2359 improved performance during the dosing period, and that these effects were sustained over the 4-week washout period. Further, there was a trend for an improvement of NS2359 over placebo for Quality of Working Memory (p < 0.1).

### 3.4 Adverse Events

No serious adverse events were reported during or following the study. There were no clinically significant changes in the measured blood and urine parameters that were evaluated to be related to the treatment. There were no clinically significant changes in the blood pressure, pulse, or the electrocardiogram evaluated to be related to the treatment. The most prominent treatment related an adverse effect reported was insomnia (Table 2). While there were more reported cases of weight reduction associated with study drug, there were no differences in rates of reported appetite suppression between groups. Most of the reported events were mild to moderate in severity.

**Table 2:** The Number and Severity of Adverse Events Experienced by the Subjects during the Study (only those events with ≥ 5% frequency are shown).

		NS2359			Placebo		
Body System	Symptom	Mild	Mod	Severe	Mild	Mod	Severe

Gastrointestinal Disorders	Diarrhea NOS	4	1	0	0	0	0
	Dry Mouth	5	3	0	7	0	0
	Dyspepsia	6	0	0	3	1	0
	Nausea	4	1	0	6	0	1
General disorders and administration site conditions	Fatigue	1	2	0	7	0	0
Infections & Infestations	Nasopharyngitis	3	1	0	4	0	1
	Upper Respiratory Tract infection	6	1	0	5	2	0
Investigations	Weight decrease	5	1	0	0	0	0
Metabolism and nutrition disorders	Appetite decreased NOS	5	1	0	6	0	0
Musculoskeletal and Connective Tissue Disorders	Back Pain	1	1	0	5	1	0
Nervous system Disorders	Dizziness	5	2	0	3	0	0
	Headache NOS	15	6	1	15	5	1
	Irregular Sleep Phases	3	0	1	2	0	0
	Somnolence	0	0	0	4	1	0
Psychiatric disorders	Initial Insomnia	1	3	0	2	0	0
	Insomnia	8	3	1	2	0	1
	Irritability	5	1	0	0	2	0
	Middle Insomnia	7	0	0	1	0	0

## Discussion

The results of this 8 week controlled study indicate that for both primary and secondary clinical outcomes, there were no clinically or statistically significant effects of NS2359 in ADHD symptoms compared to placebo for the overall study population. However, NS2359 was found to favorably affect cognitive function (the ability to focus attention and also the ability to store and retrieve episodic information) in the overall study population. A trend for improvement was also seen to working memory. These improvements are in major domains of cognitive function and have the potential for clinical relevance in ADHD patients.

Although there was little improvement compared to placebo in overall summary scores of ADHD on any of the primary or secondary outcomes, there was evidence of improvements in the inattentive subgroup with NS2359 treatment. These were seen in the patient rated ADHD-RS, the CAARS and the Brown adult scale as well as a greater reduction in the CGI-Severity of illness, a greater proportion of responders (>30% change) and a greater improvement on the CGI Improvement scale.

The NS2359 compound improved all three of the domains of cognitive function assessed with the CDR. Power of Attention reflects the ability to focus attention and to rapidly process information, and the positive effects with NS2359 indicate that subjects were better able to direct their attention to ongoing tasks and to sustain concentration. The trend for improvement in working memory indicated that the subjects were better able to hold information temporarily 'on line', potentially facilitating the performance of everyday tasks and activities. Finally the improvement to Quality of Episodic Secondary Memory with the active medication indicated that subjects were better able to encode, store and retrieve verbal and pictorial information of an episodic nature.

These cognitive data derived from the CDR testing showed that NS2359 improved attention, episodic memory, and working memory in these adults with ADHD – areas previously identified as problematic in separate studies of adults with ADHD [31]. Our combined findings of an overall lack of response coupled with evidence of improvement in the inattentive subtype and CDR suggest an overall pro-cognitive effect of the medication in ADHD. It may be that higher doses of NS2359 are required to improve the more overt behavioral symptoms of the ADHD. In support of the latter, a relationship between doses of stimulants necessary to improve cognition (lower) relative to hyperactivity/impulsivity (higher) has been speculated [32]. The lack of effect on the combined group may indicate that NS2359 has little effect on hyperactive/impulsive symptoms. An additional confounding effect could be that patients of the inattentive subtype typically have fewer ADHD symptoms and hence less severe ADHD and therefore benefit more by treatment. Given the limited previous human exposure of NS2359, a conservative low dose of the medication was chosen for safety considerations that may have under treated the entire spectrum of ADHD.

The findings of improvement in measures of cognitive functioning (CDR) and in the inattentive subtype of ADHD add to a growing literature on the usefulness of mixed catecholaminergic reuptake inhibitors [33] on ADHD. For example, studies with mixed dopaminergic and noradrenergic reuptake inhibitors such as the tricyclic antidepressants, bupropion, and noradrenergic reuptake inhibitors (atomoxetine) have yielded generally positive results, albeit with lower overall response rates and effect sizes relative to the psychostimulants. Similarly, NS2359 has been shown to enhance the release of acetylcholine in the frontal cortex and hippocampus, an effect that may be relevant for attentional performance [20,21]. For example, studies with procholinergic (nicotinic) agents have demonstrated improvement in attentional/cognitive functioning and symptoms in ADHD [16,17,34]. Hence, the combined monoaminergic reuptake inhibition plus pro-cholinergic effects of NS2359 may be related to the aggregate evidence of improved cognitive functioning in the current study.

There are a number of limitations in the current study. Because of the nature of the study, a homogenous study population was selected that may not generalize to typical adults with ADHD. For example, subjects with significant medical histories and psychiatric co-morbidities were excluded. In addition, the sample size was relatively small, limiting the power to detect differences. Furthermore, only one relatively low dose of NS2359 was studied that mostly likely underestimated the effect of the medication.

One of the dilemmas of early phase studies is the determination of the optimal dose(s) for a disorder. The dose evaluated for this study was selected on the basis of a dose-escalating study using SPECT, as well as tolerability data from pharmacokinetic studies. A dose of 0.5 mg/day NS2359 was shown to give DA occupancy of 35%, which is in the range of other clinically efficacious compounds used for treatment of ADHD patients [35]. Given only one low dose of NS2359 was tested, this dose may have been insufficient to treat ADHD.

In the current study, there were no serious side effects or reports of withdrawal symptomatology and no clinically meaningful cardiovascular or other laboratory abnormalities were detected. However the ability to detect infrequent and idiosyncratic reactions is limited by the small number of subjects and the relatively low dose and short duration of study treatment.

## Conclusion

Despite the limitations presented above, these data show that the current dose of NS2359 is well tolerated but insufficient to treat ADHD adequately in the general population of adults with ADHD. A signal emerged in the inattentive subtype and on specific measures of neuropsychological functioning suggesting the potential utility of the agent. Given the mixed findings in this group of subjects, larger, parallel design dose ranging studies with NS2359 using higher doses are warranted.

## Competing interests

Dr. Timothy Wilens receives grant support from, is a speaker for, or is a consultant for the following sources: Abbott, McNeil, Lilly, NIH (NIDA), Merck, Novartis, Cephalon and Shire.

Thorsten Klint, is a former employee of Neurosearch.

Dr. Lenard Adler receives grant support from, is a speaker for, or is a consultant for the following sources: Abbott Laboratories, Cortex Pharmaceuticals, Bristol-Myers Squibb, Merck & Co, Novartis Pharmaceuticals, Pfizer, Shire, Eli Lilly, Ortho McNeil/Jannsen/Johnson and Johnson, New River Pharmaceuticals, Cephalon, National Institute of Drug Abuse, Organon, Sanofi-Aventis Pharmaceuticals.

Dr. Scott West receives Industry Research Funding from: Abbott, Addrenex, Allergan, Allon, AstraZeneca, Avera, Bristol-Myers Squibb, Cephalon, Cortex, DOV, Eli Lilly, Epix, Forest Laboratories, GlaxoSmithKline, Jazz, Labopharm, McNeil, Merck, Myriad, Novartis, Ono, Organon, Pfizer, Saegis, Sanofi, Sepracor, Shire, Solvay, Somaxon, Transtech, Wyeth and has a consulting relationship with Abbott and Novartis.

Dr. Keith Wesnes is sole shareholder of CDR Ltd, which received financial support to supply the cognitive tests in the study. His company is employed by dozens of pharmaceutical companies and he is a consultant to a number of companies also.

Ole Graff, is an employee of GSK and former employee at Neurosearch.

Birgit Ohrt Mikkelsen is an employee of NeuroSearch and owns NeuroSearch shares.

## Authors' contributions

TW, TK, LA, OG, and BM assisted in the design of the study, TW, LA, and SW executed the study, KW was involved in the neuropsychological assessments, and all authors were involved in the development and editing of the final manuscript.
